# Supplementary material for: End Sequence Analysis Toolkit (ESAT) expands the extractable information from single-cell RNA-seq data
Source: Genome Res. 2016 Oct;26(10):1397–410. doi: 10.1101/gr.207902.116 (PMC5052061; doi:10.1101/gr.207902.116)
Supplement: Supplemental Material [file supp_gr.207902.116_Supp_Materials.tar.gz › ESAT-esat_v0.1/doc/html/interfaceumms_1_1core_1_1feature_1_1_window.html]

ESAT: umms.core.feature.Window Interface Reference


ESAT


- Main Page
- Related Pages
- Packages
- Classes
- Files

- Class List
- Class Index
- Class Hierarchy
- Class Members

All Classes Namespaces Files Functions Variables Enumerator Pages

- umms
- core
- feature
- Window

Public Member Functions |
List of all members

umms.core.feature.Window Interface Reference

Inheritance diagram for umms.core.feature.Window:

[legend]

Collaboration diagram for umms.core.feature.Window:

[legend]

|  |  |
| --- | --- |
| Public Member Functions | |
| void | addSourceAnnotation (Annotation annotation) |
|  | |
| Collection<?extends Annotation > | getSourceAnnotations () |
|  | |
| Collection< Window > | getWindows (int windowSize, int stepSize) |
|  | |
| Public Member Functions inherited from umms.core.annotation.Annotation | |
| int | getStart () |
|  | |
| int | getSAMStart () |
|  | |
| int | getEnd () |
|  | |
| int | getSAMEnd () |
|  | |
| String | getReferenceName () |
|  | |
| String | getChr () |
|  | |
| String | getName () |
|  | |
| Strand | getOrientation () |
|  | |
| Strand | getStrand () |
|  | |
| boolean | hasOrientation () |
|  | |
| boolean | isNegativeStrand () |
|  | |
| int | numBlocks () |
|  | |
| List<?extends Annotation > | getBlocks () |
|  | |
| List<?extends Annotation > | getBlocks (boolean oriented) |
|  | |
| int | length () |
|  | |
| int | getSize () |
|  | |
| int | size () |
|  | |
| int | getLengthOnReference () |
|  | |
| int | getOrientedStart () |
|  | |
| int | getOrientedEnd () |
|  | |
| double | getScore () |
|  | |
| boolean | isUnoriented () |
|  | |
| int | getReferenceCoordinateAtPosition (int positionInAnnotation) |
|  | |
| int | getPositionAtReferenceCoordinate (int referenceCoordinate) |
|  | |
| int | getReferenceCoordinateAtPosition (int positionInAnnotation, boolean ignoreOrientation) |
|  | |
| int | getPositionAtReferenceCoordinate (int referenceCoordinate, boolean ignoreOrientation) |
|  | |
| void | setStart (int start) |
|  | |
| void | setEnd (int end) |
|  | |
| void | setOrientation (char orientation) |
|  | |
| void | setOrientation (Strand orientation) |
|  | |
| void | setOrientedStart (int orientedStart) |
|  | |
| void | setOrientedEnd (int orientedEnd) |
|  | |
| void | setReferenceName (String refName) |
|  | |
| void | setName (String name) |
|  | |
| void | setScore (double score) |
|  | |
| boolean | equals (Annotation other) |
|  | |
| void | expand (int deltaStart, int deltaEnd) |
|  | |
| Annotation | trim (int deltaStart, int deltaEnd) |
|  | |
| void | shift (int delta) |
|  | |
| void | moveToCoordinate (int coordinateInReference) |
|  | |
| Annotation | copy () |
|  | |
| List< Annotation > | disect (Annotation a) |
|  | |
| List< Annotation > | disect (List<?extends Annotation > disectors) |
|  | |
| Annotation | minus (Annotation other) |
|  | |
| Annotation | minus (Collection<?extends Annotation > others) |
|  | |
| int | getDistanceTo (Annotation other) |
|  | |
| String | toUCSC () |
|  | |
| String | toBED () |
|  | |
| String | toBED (int r, int g, int b) |
|  | |
| String | toShortBED () |
|  | |
| String | toBEDGraph () |
|  | |
| String | getFullInfoString () |
|  | |
| boolean | overlaps (Annotation other, int buffer) |
|  | |
| boolean | overlaps (Collection<?extends Annotation > others, int buffer) |
|  | |
| boolean | overlaps (Annotation other) |
|  | |
| boolean | overlaps (Collection<?extends Annotation > others) |
|  | |
| boolean | overlaps (Annotation other, int buffer, boolean considerOrientation) |
|  | |
| boolean | overlaps (Annotation other, boolean considerOrientation) |
|  | |
| boolean | overlapsStranded (Annotation other) |
|  | |
| int | getOverlap (Annotation other) |
|  | |
| boolean | contains (Annotation other) |
|  | |
| Annotation | union (Annotation other) |
|  | |
| Annotation | intersect (Annotation other) |
|  | |
| List< Annotation > | intersect (List<?extends Annotation > annotations) |
|  | |
| int | compareToAnnotation (Annotation b) |
|  | |
| void | stitchTo (Annotation next) |
|  | |
| String | toCigar () |
|  | |
| boolean | fullyContains (Annotation annotation) |
|  | |
| Annotation | complement () |
|  | |
| boolean | equals (Annotation other, boolean useOrientation) |
|  | |
| Collection<?extends Annotation > | getSpliceConnections () |
|  | |
| int | getMidpoint () |
|  | |

## Member Function Documentation

|  |  |  |  |  |  |
| --- | --- | --- | --- | --- | --- |
| void umms.core.feature.Window.addSourceAnnotation | ( | Annotation | *annotation* | ) |  |

Add a pointer to the annotation from which this Window originated

Parameters
:   |  |  |
    | --- | --- |
    | annotation |  |

Implemented in umms.core.feature.GeneWindow, and umms.core.feature.GenomeWindow.

|  |  |  |  |  |
| --- | --- | --- | --- | --- |
| Collection<? extends Annotation> umms.core.feature.Window.getSourceAnnotations | ( |  | ) |  |

Returns
:   a collection of annotations from which this originated

Implemented in umms.core.feature.GeneWindow, and umms.core.feature.GenomeWindow.

|  |  |  |  |
| --- | --- | --- | --- |
| Collection<Window> umms.core.feature.Window.getWindows | ( | int | *windowSize*, |
|  |  | int | *stepSize* |
|  | ) |  |  |

Get collection of windows spanning the annotation

Parameters
:   |  |  |
    | --- | --- |
    | windowSize | Window size |
    | stepSize | Step size |

Returns
:   The collection of windows

Implemented in umms.core.feature.GeneWindow, and umms.core.feature.GenomeWindow.

---

The documentation for this interface was generated from the following file:

- java/umms/core/feature/Window.java


---

Generated on Fri Aug 15 2014 15:11:10 for ESAT by  

 1.8.7
